# Supplementary material for: China’s non-ferrous metal recycling technology convergence and driving factors: A quadratic assignment procedure analysis based on patent collaboration-based network structural hole
Source: PLoS One. 2022 Aug 5;17(8):e0271694. doi: 10.1371/journal.pone.0271694 (PMC9355239; doi:10.1371/journal.pone.0271694)
Supplement: Attachment — Submitted filename: Review -01.pdf [file pone.0271694.s001.pdf]

This paper presents the impact of structural holes in inter-organizational technology cooperation networks on technology convergence from a patent perspective in China's non–ferrous metal resource recycling technology as a case study. Although the concept is nice but it requires a few modifications to further improve the quality.

1- The introduction part is well researched but the author/s should clearly emphasize the advantages of the methods used in this study, Introducing the methods alone is not enough and the necessity of choosing them is also important.

2- In data-driven research, the methodology of data analysis and information about the data used in the research are important. In the introduction section, The author/s are more concerned about the application, rather than the data and methodologies.

3- Authors should provide a general figure of the framework of the research at the end of the introduction section in full detail.

4- The Results (section 4.) and Research method (sub-section 3.3) text part are very weak. It is true that the methodology is not innovative and is known, but a more accurate introduction of the methods is needed. The reader does not have any clear information about how the methods work. Clear articulation of methods and results in light of the Research question or hypothesis is needed.

5- Results:  I suggest you discuss a little bit more the results of the case study with the previous (and recent) literature. I think you have space here to discuss more your results and findings.
